# Controlling emerging zoonoses at the animal-human interface

**DOI:** 10.1186/s42522-020-00024-5

**Published:** 2020-09-18

**Authors:** Riley O. Mummah, Nicole A. Hoff, Anne W. Rimoin, James O. Lloyd-Smith

**Affiliations:** 1 Department of Ecology and Evolutionary Biology, University of California, 610 Charles E Young Dr S, Los Angeles, CA 90095 USA; 2 Department of Epidemiology, University of California, Los Angeles, CA 90095 USA; 3 Fogarty International Center, National Institutes of Health, Bethesda, MD 20892 USA

**Keywords:** Subcritical zoonoses, Stuttering zoonoses, Epidemiological control, Emerging infectious diseases, Cross-species spillover transmission, Human-to-human transmission, Infectious disease dynamics

## Abstract

**Background:**

For many emerging or re-emerging pathogens, cases in humans arise from a mixture of introductions (via zoonotic spillover from animal reservoirs or geographic spillover from endemic regions) and secondary human-to-human transmission. Interventions aiming to reduce incidence of these infections can be focused on preventing spillover or reducing human-to-human transmission, or sometimes both at once, and typically are governed by resource constraints that require policymakers to make choices. Despite increasing emphasis on using mathematical models to inform disease control policies, little attention has been paid to guiding rational disease control at the animal-human interface.

**Methods:**

We introduce a modeling framework to analyze the impacts of different disease control policies, focusing on pathogens exhibiting subcritical transmission among humans (i.e. pathogens that cannot establish sustained human-to-human transmission). We quantify the relative effectiveness of measures to reduce spillover (e.g. reducing contact with animal hosts), human-to-human transmission (e.g. case isolation), or both at once (e.g. vaccination), across a range of epidemiological contexts.

**Results:**

We provide guidelines for choosing which mode of control to prioritize in different epidemiological scenarios and considering different levels of resource and relative costs. We contextualize our analysis with current zoonotic pathogens and other subcritical pathogens, such as post-elimination measles, and control policies that have been applied.

**Conclusions:**

Our work provides a model-based, theoretical foundation to understand and guide policy for subcritical zoonoses, integrating across disciplinary and species boundaries in a manner consistent with One Health principles.

## Background

Zoonotic pathogens are a major threat to global health, both through their on-going contributions to disease burden and their potential contributions to the emergence of novel pandemic pathogens [1]. Zoonotic spillover is defined as transmission of a pathogen from an animal host to a susceptible human and is the source of diseases from monkeypox to plague to leishmaniasis. Risk of zoonotic spillover is driven by many ecological, epidemiological, and behavioral factors across scales [2–4]. The combination of animal ecology, human behavior, and environmental conditions can lead to cross-species transmission and, thus, requires a OneHealth perspective to evaluate and respond to outbreaks of disease.

Beyond the complexity of the zoonotic spillover process, zoonotic pathogens differ greatly with respect to their efficiency of human-to-human transmission [5, 6]. Transmissibility between humans is described by the reproductive number, *R*_0_, which is defined as the average number of secondary cases caused by a single infected individual in an entirely susceptible population [7]. Some zoonotic pathogens face pre-existing immunity in the population, and are governed by the effective reproductive number, *R*_eff_, which is the average number of secondary cases in a population with both susceptible and immune individuals [8–10]. For simplicity, we will use *R* throughout this study to refer to *R*_0_ or *R*_eff_, in either case representing the efficiency of human-to-human transmission before further control measures are considered.

It is useful to classify zoonoses by their transmissibility among humans, as captured by their *R* value [6]. For pathogens with *R* = 0, like West Nile virus or rabies virus, transmission only occurs through spillover, and the pathogen is unable to transmit between humans. When *R* is between 0 and 1, the pathogen is subcritical and causes self-limiting outbreaks, as for monkeypox virus, Nipah virus, or some avian influenza viruses. Supercritical pathogens with *R* > 1, such as pandemic influenza or Ebola virus, can cause epidemics or pandemics in the human population.

Subcritical zoonoses have been understudied by infectious disease modelers, likely because they do not align with dominant modeling frameworks, they require integration of animal and human dynamics, and they presently lack pandemic potential [6]. Modeling effort on these systems has been particularly sparse for questions of disease control and its economic components, and the interplay between spillover and human-to-human transmission in driving the epidemiology of subcritical pathogens. Some improvements have been made in the last decade, especially in methods for estimating *R* for subcritical pathogens [11–15], but there is still a lack of theory to guide control efforts [16]. This paper aims to address this gap. We note that our findings extend directly to non-zoonotic pathogens such as post-elimination measles, where *R* <  1 due to herd immunity, and geographic importation plays the role of spillover. Our findings also relate to the on-going COVID-19 pandemic caused by SARS-CoV-2, where *R* <  1 in some settings due to social distancing, contact tracing, quarantine and isolation, and other measures, and again geographic importation acts like spillover to introduce new cases. Our work also applies equally to pathogens that transmit directly or via arthropod vectors.

**Fig. 1:**
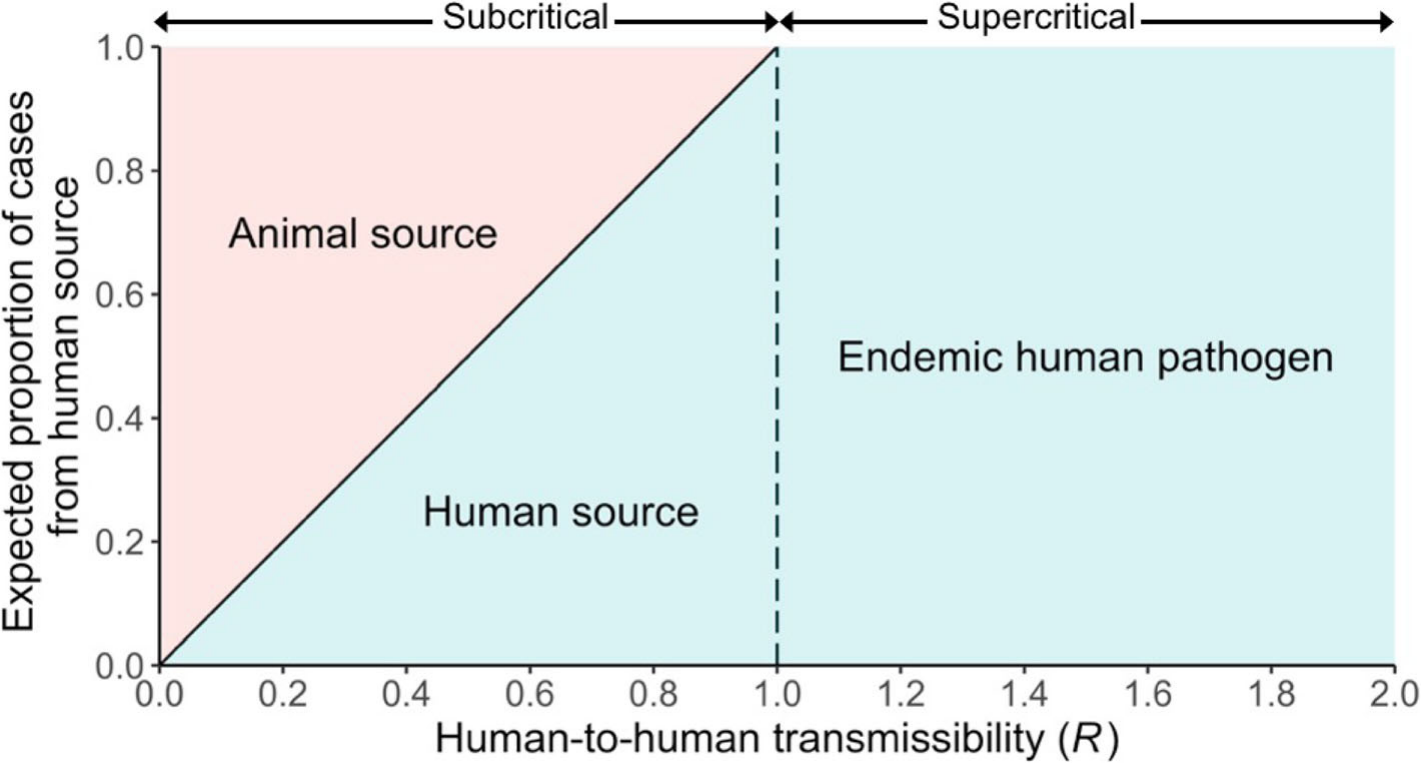
The expected source of infection for cases is determined by the reproductive number for human-to-human transmission

Control measures for subcritical zoonoses can be classified into three functional groups according to the modes of transmission they aim to reduce: prevention of spillover, reduction of human-to-human transmission, and control of both spillover and human-to-human transmission jointly (Table 1). Because subcritical pathogens cannot cause epidemics and every outbreak is triggered by a spillover event, public health policy may naturally focus on spillover prevention. However as *R* rises toward 1, an increasing proportion of cases are caused by human-to-human transmission (Fig. 1). This leads to open questions about how to target control measures. Should control be targeted at animal-to-human transmission, human-to-human transmission, or both? Furthermore, for pathogens that spill over infrequently, implementing controls that focus on human-to-human transmission when there are no active outbreaks does not seem cost effective. Would a reactive strategy which switches from preventing zoonotic spillover to reducing human-to-human transmission be more effective?

**Table 1:** A collection of systems which have implemented control measures targeted at spillover animal-to-human transmission, human-to-human transmission, or both and their associated reproductive numbers

**Pathogen**	***R***	**Transmission mode targeted**	**Intervention**	**References**
**Subcritical pathogens**				
Avian influenza (H7N9)	0.06 to 0.35 [17]	Spillover	Reductions in poultry exports Market disinfection Closure of live poultry trading activities	[17–21]
		Human-to-human	Handwashing Social distancing	
		Both	Poultry trade regulations Health education campaigns	
Avian influenza (H5N1)	0.05 to 0.2 [22]	Spillover	Culling poultry	[23, 24]
		Human-to-human	Active case surveillance	
		Both	Vaccination	
Nipah	0.48 [25]	Spillover	Avoid consumption of palm sap Covers for palm sap collection vessels	[26]
		Human-to-human	Nosocomial interventions	
Monkeypox	~ 0.3 in 1980s [11]	Spillover	Reduce contact with reservoir animals	[27–30]
		Human-to-human	Improved diagnostics for early case detection Vaccinating healthcare workers	
		Both	Smallpox vaccination Community-based interventions	
MERS	0.45 [12]	Spillover	Reduction of human contact with camels Camel vaccination	[12, 31–33]
		Human-to-human	Nosocomial precautions Case isolation	
Lassa	0.73 [34]	Spillover	Rodent control	[34–38]
		Human-to-human	Nosocomial precautions (PPE) Contact precautions (household and hospital settings)	
Post-elimination measles	0.45 [39]	Spillover (reintroduction)	Targeted vaccination of international travelers	[39–41]
		Both	Vaccination	
Crimean-Congo hemorrhagic fever (CCHF)	< 1^a^	Human-to-human (nosocomial)	Case isolation Contact precautions (PPE)	[42–44]
**Supercritical pathogens**				
SARS-CoV	2.7 [45]	Spillover	Culling civets Reducing contacts in live markets	[45, 46]
		Human-to-human	Quarantine Case isolation Contact precautions (PPE)	
SARS-CoV-2	2.2–6.47 [47, 48]	Spillover	Market closure	[49, 50]
		Human-to-human	Social distancing Travel restrictions Contact precautions (PPE) Quarantine and isolation	
Ebola	1.5 to 2.5 [51–53]	Spillover	Avoid contact with animals found dead Vaccination of nonhuman primates	[51, 54–59]
		Human-to-human	Safe burial Contact precautions (PPE) Drug treatments Ring vaccination	
		Both	Vaccination of general population	
Yellow fever	4.8 [62]	Spillover	Vector control	[60, 61]
		Human-to-human	Urban vector control	
		Both	Vaccination Protective behavior (PPE)	

^a^ Human-to-human transmission has been reported in nosocomial settings but is rare

This decision space gets more complicated when economic costs of control are considered, especially because measures to reduce different types of transmission may differ in cost. Similarly, there are often known host or environmental factors that influence spillover risk. Epidemiological risk factor studies can define these factors so high-risk groups can be identified. For example, individuals in contact with dromedary camels and hunting or handling bushmeat are at higher risk for transmission of Middle East Respiratory Syndrome (MERS) coronavirus and simian retroviruses, respectively [63, 64]. Can targeted reduction of spillover in these high-risk groups be an effective control measure for subcritical pathogens? This study presents a general theory to build intuition and give evidence-based guidelines for effective control of subcritical zoonoses (or other subcritical pathogens). Our framework reveals general principles to aid policymakers faced with difficult decisions and resource constraints and can be adapted to specific pathogens and settings to guide concrete decisions and support allocation of finite resources.

## Methods

### Total incidence of subcritical zoonoses

For a subcritical zoonosis, the total incidence of infection in the human population arises from a mixture of primary and secondary cases. We assume that zoonotic spillover events (primary cases) occur at some characteristic total rate *λ*_*z*_ in a population of interest, e.g. *λ*_*z*_ might represent 100 spillover events per year in a given administrative region. Each human case is then capable of transmitting the infection to cause secondary cases, with the reproductive number *R* denoting the expected number of secondary cases per infected human.

It is possible to model the stochastic dynamics of transmission in the human population using a branching process [65–67] or similar formulation. For the present analysis, it is sufficient to focus on the expected dynamics of these models. The first generation of transmission (i.e. those infected by the index case) will have *R* cases, on average. Each of these cases will infect another *R* cases, so the second generation will have *R*^*2*^ cases, and so on. Thus the mean number of cases in each minor outbreak, including the primary case that triggers the outbreak, is given by a geometric series, and for *R* < 1 [68]:

1 $$ {\displaystyle \begin{array}{c}E\left( total\kern0.5em \#\kern0.5em of\kern0.5em cases\kern0.5em per\kern0.5em outbreak\right)=1+R+{R}^2+{R}^3+\cdots \\ {}=\frac{1}{1-R}\end{array}} $$

The expected total incidence rate *I* is then given by the product of the spillover rate and the mean number of cases associated with each spillover event:

2 $$ I=\frac{\lambda_z}{1-\mathrm{R}}. $$

### Proportion of cases infected by human sources

As shown above (Eq. 1), when *R* < 1, the expected number of cases that result from each introduction is 1/(1-*R*). Because there is one primary case per introduction, the proportion of cases that are primary is given by the reciprocal of this quantity and is equal to (1-*R*). Thus, for zoonotic pathogens with *R* < 1, the expected proportion of cases infected by humans is *R*.

### Analysis of control measures for stuttering zoonoses

We consider the effect on total incidence of control measures that reduce spillover rates, human-to-human transmission, or both. Let *c*_*z*_ be the factor by which control measures reduce spillover rates, and *c*_*R*_ be the factor by which control measures reduce the reproductive number in the human population. When these reduction factors equal 1, there is no impact on transmission; when they equal 0, that mode of transmission is halted completely. The total incidence under control, *I*_*c*_, is then:

3 $$ {I}_c=\frac{c_z{\lambda}_z}{1-{c}_RR} $$

For control measures that affect both modes of transmission equally, such as protective vaccination of humans, *c*_*z*_ = *c*_*R*_ = *c* and this expression simplifies accordingly.

In settings where it is necessary to choose between measures to reduce zoonotic spillover (via *c*_*z*_) and measures to reduce human-to-human transmission (via *c*_*R*_), we can compare the total incidence when each measure is in place. The point where the two strategies are equivalent is given by:

4 $$ \frac{\lambda_z}{1-{c}_RR}=\frac{c_z{\lambda}_z}{1-R} $$

Since the zoonotic spillover rate enters both sides linearly, the preferred strategy in a given context is governed by the reproductive number. Rearranging these expressions, we can find the value of the reproductive number where the two strategies are equivalent:

5 $$ {R}_{switch}=\frac{1-{c}_z}{1-{c}_z{c}_R} $$

When *R > R*_*switch*_, the strategy to reduce human-to-human transmission will yield a greater reduction in incidence.

### Cost-benefit analysis of control measures

**Fig. 2:**
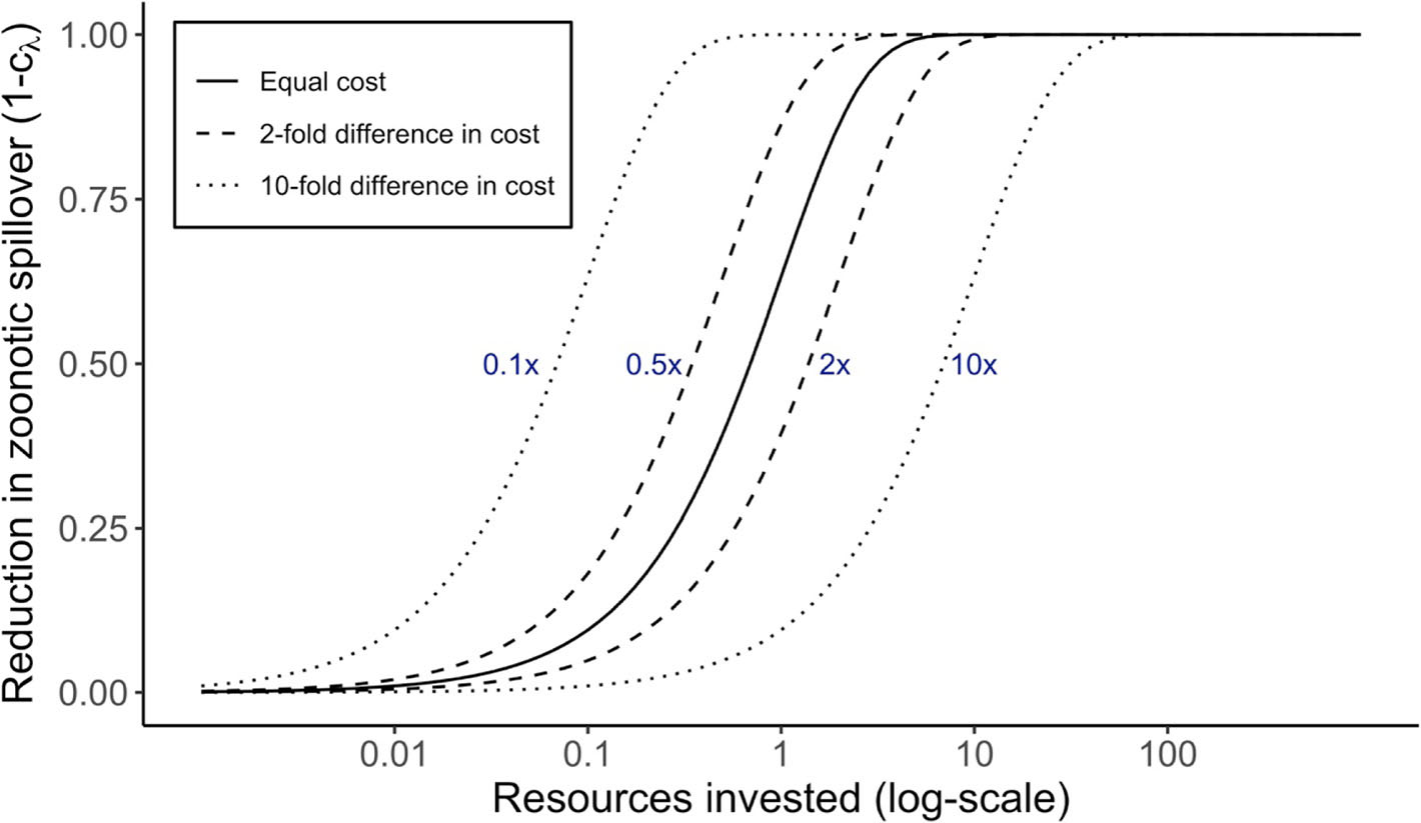
The cost function for control measures. The solid black line indicates equal cost between implementing spillover control and reducing human-to-human transmission. The x-axis shows an arbitrary scale of resource investment. The dashed and dotted lines show a 2-fold and 10-fold difference, respectively, in the costs of the two control measures. Each line is marked by the relative cost of reducing spillover by a given proportion compared to the cost of reducing human-to-human transmission by the same proportion

To incorporate the influence of differing cost, we consider how the effectiveness of control measures varies with effort using a simple model for the principle of diminishing returns on investment (Fig. 2). This corresponds to many public health settings where heterogeneity in the structure, accessibility and compliance of populations means that the incremental cost of expanding coverage rises as coverage rises. We model this phenomenon by setting the effectiveness of control measures to be a declining exponential function of resources invested, *r*. To reflect the different costs of different control strategies, we introduce a factor α to scale the return on investment for reducing spillover relative to reducing human-to-human transmission:

6 $$ {c}_z={e}^{-\alpha r}\  and\ {c}_R={e}^{-r} $$

Substituting these expressions into Eq. 5 and solving for the value of the reproductive number *R*_*switch*_ at which the strategies are equivalent, we find:

7 $$ {R}_{switch}=\frac{1-{e}^{-\alpha r}}{1-{e}^{-\left(\alpha +1\right)r}} $$

**Fig. 3:**
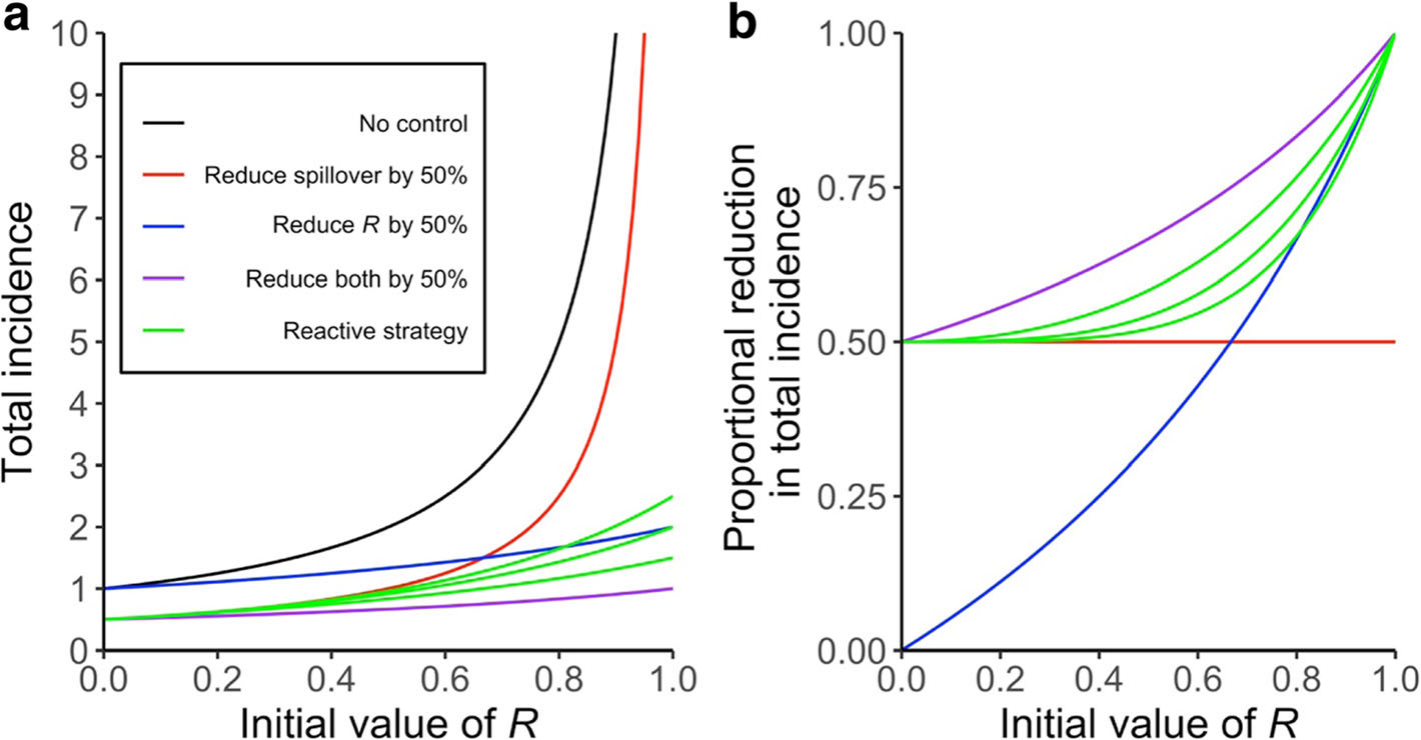
Impacts of different control measures on incidence of a zoonotic infection with initial value of R (i.e. before control) between 0 and 1. Panel **a** illustrates the effects of control on the total incidence expected in a focal population, whereas panel **b** shows the proportional reduction in expected incidence when compared to the incidence level without control (black line). In **a**, the black line shows how the expected total incidence increases nonlinearly with *R*, for a fixed rate of zoonotic spillover. Colored lines show the total incidence that would result from interventions that cause 50% reductions in spillover transmission (red), human-to-human transmission (blue), or both types of transmission (purple). Green lines show the incidence resulting from a reactive intervention strategy, where effort is focused on reducing spillover transmission but is shifted to reducing human-to-human transmission once an outbreak is detected. The three green lines show the total incidence resulting when control is shifted after one, two, or three generations of transmission among humans, respectively from top to bottom

*R*_*switch*_ is the value of *R* above which control measures should be targeted at human-to-human transmission, for a given level of resource investment. The curves in Fig. 3 are generated by plotting 1 – *c*_*z*_ (or equivalently, 1 − *e*^−*αr*^) versus *R*_*switch*_ parametrically as a function of *r*, for different values of the relative cost α (which are shown as different line types).

### Analysis of reactive control measures

For a given reduction factor (*c*) describing the effectiveness of control, the greatest reduction in incidence is obtained from control measures such as vaccination that simultaneously reduce both zoonotic spillover and human-to-human transmission. When this is not possible and when resources are constraining, it may be desirable to implement control measures in a reactive manner. Here we analyze the impact of control policies that focus on reducing spillover as a default but switch their focus to reducing human-to-human transmission when an outbreak has been detected. We assume that the switch occurs after *k* generations of transmission among humans, reflecting inevitable delays in case identification and policy implementation. If control is imposed after the first generation of human-to-human transmission has occurred (*k* = 1), then the first generation will have expected size *R* and subsequent generations will have expected size *c*_*R*_*R*. Thus, the expected number of cases in the minor outbreak will be:

8 $$ {\displaystyle \begin{array}{c}E(X)=1+R+R\left({c}_RR\right)+R{\left({c}_RR\right)}^2+\cdots \\ {}=\frac{1}{c_R}\left(\frac{1}{1-{c}_RR}\right)+\left(1-\frac{1}{c_R}\right)\end{array}} $$

In the Supplement we show that the general form, for a delay of *k* generations, will be:

9 $$ E(X)=\frac{1}{c_R^k}\ \left(\frac{1}{1-{c}_RR}\right)+\sum \limits_{i=0}^k{R}^i\left(1-\frac{c_R^i}{c_R^k}\right) $$

Thus the total incidence under reactive control strategy, with reduction factors *c*_*R*_ and *c*_*z*_, is:

10 $$ {I}_c={c}_z{\lambda}_z\left(\frac{1}{c_R^k}\ \left(\frac{1}{1-{c}_RR}\right)+\sum \limits_{i=0}^k{R}^i\left(1-\frac{c_R^i}{c_R^k}\right)\right) $$

This expression was used to plot the green curves in Fig. 3 with *c*_*R*_ = *c*_*z*_.

### Decomposing spillover rate into risk groups

For most zoonotic pathogens, zoonotic spillover risk is not fixed across the population due to variation in ecological, epidemiological, and behavioral factors. To explore how heterogeneous spillover risk might influence the choice of disease control strategy, we analyze a model with two defined groups with different risk of zoonotic infection. We define *p* as the proportion of the population in the group with higher risk of zoonotic spillover. Let *λ*_*H*_ be the spillover rate for the high-risk group and *λ*_*L*_ be the spillover rate for the low risk group. Then the total spillover rate *λ*_*z*_ can be written as *pλ*_*H*_ + (1 − *p*)*λ*_*L*_. Thus, in the absence of control measures, the incidence is given by:

11 $$ I=\frac{p{\lambda}_H+\left(1-p\right){\lambda}_L}{1-R} $$

### Analysis of control measures for subcritical zoonoses with risk groups

We now consider the effect of different control measures when there are defined high-risk and low-risk spillover groups. Like before, we can apply a general spillover reduction term, *c*_*λ*_, that applies to both risk groups to reduce the two spillover rates equally. The total incidence is then:

12 $$ {I}_c=\frac{c_{\lambda}\left(p{\lambda}_H+\left(1-p\right){\lambda}_L\right)}{1-{c}_RR} $$

In settings with defined risk groups, interventions that target the high-risk group are an attractive strategy for reducing overall incidence. To model control measures targeted at the high-risk spillover group, we define *c*_*H*_ to be the factor by which control measures reduce spillover rates in the high-risk group. The total incidence under such a targeted control policy is then:

13 $$ {I}_c=\frac{c_Hp{\lambda}_H+\left(1-p\right){\lambda}_L}{1-{c}_RR} $$

We assume that targeted control makes more efficient use of resources, in proportion to the size of the high-risk group. Thus to calculate the effect of targeted control in our cost-benefit analyses, we multiply the resources invested by a factor *1/p*. The reduction factor *c*_*H*_ is thus a function of resources invested, *r*, the factor α that reflects the relative cost of reducing spillover versus reducing human-to-human transmission, and the proportion of high-risk individuals in the population, *p*:

14 $$ {c}_H={e}^{-\alpha r/p} $$

Different combinations of targeted spillover reduction, universal spillover reduction, and efforts to reduce *R* give rise to incidence expressions similar to Eqs. (10) and (13). Table S1 contains the full list of equations that were used to plot the curves in Figs. 6 and 7.

## Results

### Incidence and control of subcritical zoonoses

Our analysis of control measures for subcritical zoonoses is guided by strikingly simple predictions, derived from basic theory for outbreak dynamics, about the expected proportion of all human cases infected by other humans versus by animals (Fig. 1; Eq. 1). The relative importance of these transmission routes in any system is governed by the efficiency of human-to-human transmission, as quantified by *R*. For subcritical zoonoses with *R* < 1, the expected number of cases that result from each introduction (including the 1 primary case) is 1/(1–*R*), and thus the proportion of cases infected by humans is *R*. When *R* > 1, endemic circulation of the pathogen in the human population is possible. Averaging over many introductions, secondary cases from successful outbreaks greatly outweigh the primary cases, including instances where introductions go extinct, and effectively all cases are from human sources.

For a given rate of zoonotic spillover, the expected total incidence level depends strongly on the prevailing value of *R*, with expected outbreak sizes rising sharply as *R* approaches 1 (Fig. 3a). Accordingly, disease control interventions exhibit marked differences in effectiveness as a function of *R*, in both absolute and proportional terms (Fig. 3a,b). Because the total incidence scales linearly with the spillover rate (Eq. 2), measures that reduce spillover transmission have a fixed proportional impact, regardless of *R* (Fig. 3b). Measures to reduce human-to-human transmission have limited impact when *R* is low, compared to measures reducing zoonotic spillover, but this situation is reversed dramatically as *R* approaches 1 (Fig. 3). When comparing measures that reduce either type of transmission by 50% (i.e. comparing *c*_*z*_ = 0.5 to *c*_*R*_ = 0.5), spillover-reducing measures are preferred for *R* values up to 0.67, then measures reducing human-to-human transmission are preferred above this point (Eq. 5, Fig. 3a). The most effective control measures are those, like vaccination, that reduce both routes of transmission by a given amount. When vaccines are not available, as is initially the case for many emerging pathogens, a reactive strategy that targets spillover then switches to human-to-human transmission once an outbreak is detected can be almost as effective, even if the switch is delayed for several generations of transmission (Fig. 3a,b).

### Optimal control strategies with different levels of resources

**Fig. 4:**
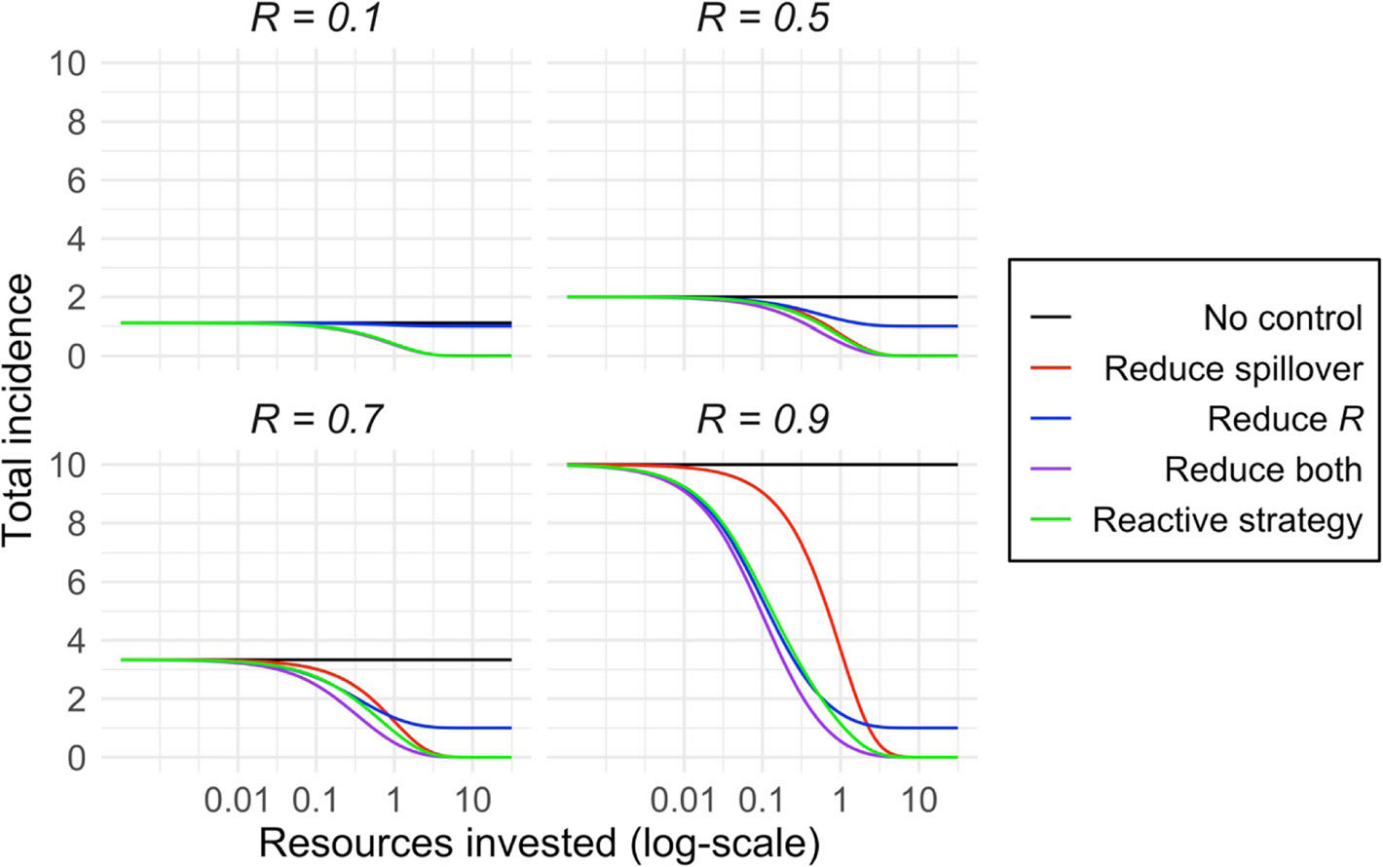
Impacts of control measures with varying resource investments on incidence of a zoonotic infection with *R* between 0 and 1. Each panel shows a different *R* (before control) value. The black lines show the incidence under no control. Colored lines show the change in total incidence that would result from increasing investment for interventions that cause reductions in spillover transmission (red), human-to-human transmission (blue), or both types of transmission (purple). The green line shows the incidence for increasing investment resulting from a reactive intervention strategy, where effort is focused on reducing spillover transmission but is shifted to reducing human-to-human transmission once an outbreak is detected (Detection after two generations of transmission is shown). Controls measures targeting spillover transmission are assumed to be equally costly as measures targeting human-to-human transmission, i.e. α = 1

We then considered how various control strategies perform under different scenarios of resource investment, where resources govern the effectiveness of control measures via our assumption of diminishing returns on investment (Fig. 2). Two findings stood out. First, control measures that focus strictly on reducing human-to-human transmission will never reduce incidence to zero (Fig. 4). Even with very high resource investment, when human-to-human transmission is halted entirely, primary cases are undiminished. However, at lower resource levels, measures to reduce human-to-human transmission can be cost-effective, depending on the epidemiological context (Fig. 4). In low transmissibility settings, investing resources into reducing human-to-human transmission is only barely better than doing nothing. As *R* increases, though, we see a growing range of resource levels where reducing *R* is more effective than reducing spillover. When *R* = 0.9, this difference is large and persists throughout almost the full range of incidence reduction – yet only spillover reduction can drive incidence to zero (Fig. 4). Unsurprisingly, if it is possible to reduce both modes of transmission at the same cost as reducing one, then this is always the most cost-effective strategy. Notably, though, the reactive strategy is nearly as cost-effective, given the costs of reducing cross-species transmission and human-to-human transmission are equal.

**Fig. 5:**
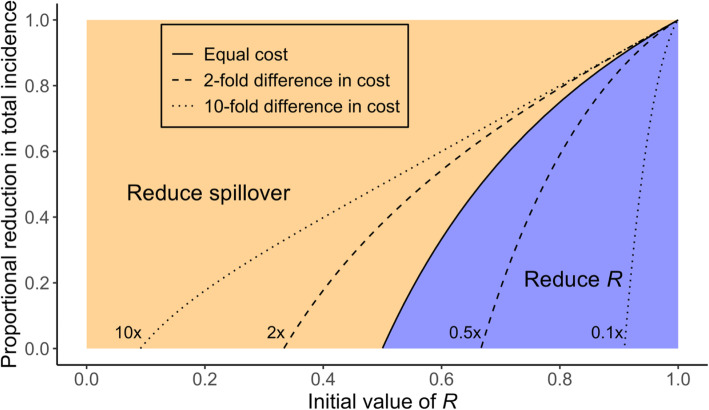
Policy guidance whether incidence will be reduced more by focusing on reducing spillover transmission or human-to-human transmission, for different values of *R* (before control) and the reduction in spillover that is achievable given resource constraints. The solid line shows the boundary between preferred strategies when costs of the two types of control are equal, as defined by Eq. (5). The dashed and dotted lines show how the boundary shifts due to differences in relative cost (each line is labeled by the relative cost of reducing spillover by a given proportion compared to the cost of reducing *R* by the same proportion)

For most emerging zoonoses a vaccine is unavailable, and in many settings reactive measures may not be practical due to logistical challenges, unavoidable delays, or shortcomings in surveillance. In these settings a choice must be made between reducing zoonotic spillover or reducing human-to-human transmission, and we can determine which strategy would be most effective for a given *R* value. The preferred strategy depends on the level of resources available, which we quantify here by the proportional reduction in spillover that is achievable if all resources are devoted to spillover control (Fig. 5). The curved solid line marks the boundary between optimal strategies, assuming it is equally expensive to implement spillover reduction or human-to-human transmission reduction (i.e. α = 1). This line corresponds to Eq. 7 for *R*_*switch*_, plotted parametrically as a function of resource investment (i.e. with zero investment and *c*_*z*_ = 1 at the bottom, and infinite investment and *c*_*z*_ = 0 at the top). When *R* is low or resource levels are high (Fig. 5 - shaded in orange), it is preferable to cut off zoonoses at the source by focusing control efforts on reducing cross-species spillover. As *R* approaches 1, it becomes more effective to reduce human-to-human transmission, as a diminishing fraction of cases are attributed to spillover. Yet in order to drive total incidence to zero, in the limit of high resource investment, it is necessary to focus on spillover reduction. If the costs of the strategies are not equal, the tradeoff line shifts (Fig. 5, dotted and dashed lines). The greater the cost of reducing spillover transmission relative to reducing human-to-human transmission, the more the tradeoff curve moves to the left, indicating that targeting human-to-human transmission would be a better use of resources for lower *R* values – though spillover reduction always becomes preferable as we aim to push incidence levels toward zero (Figs. 4 and 5). Conversely, if reducing spillover transmission is substantially cheaper than reducing human-to-human spread, then spillover reduction remains the preferred strategy for values of *R* approaching 1 (Fig. 5).

### Risk heterogeneity and the potential benefits of targeted control

**Fig. 6:**
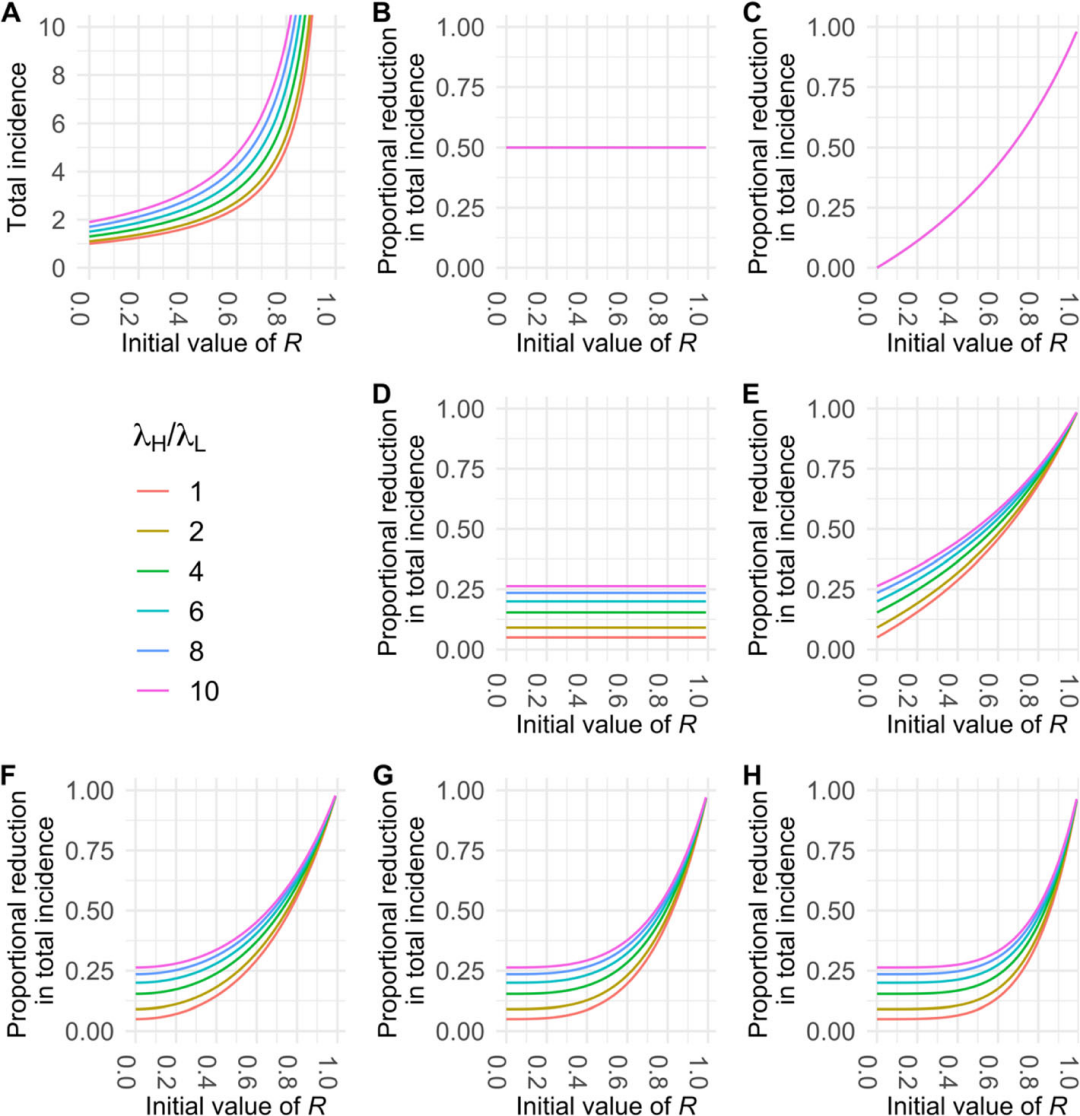
Impacts of different control measures on the total incidence of a zoonotic infection with *R* (before control) between 0 and 1 with a varying ratio of high and low spillover rates. Panel **a** shows how total incidence increases with *R* for varying ratios of high-to-low zoonotic spillover. Panels **b**-**e** illustrate proportional reduction in incidence for controls that would cause 50% reductions in all spillover transmission (**b**), human-to-human transmission (**c**), spillover transmission into the high-risk group (**d**), or jointly high-risk spillover and human-to-human transmission (**e**). Panels **f**-**h** show the proportional reduction in incidence given a reactive strategy that first targets high-risk spillover and then switches to reducing human-to-human transmission after 1, 2, or 3 generations of transmission, respectively. Note that longer delays cause the results to resemble Panel **d** over increasing ranges of *R* values, since at low *R* many transmission chains don’t last multiple generations. The proportion of high-risk individuals in the population was set to 0.10

In many settings, there are identifiable groups at elevated risk for zoonotic spillover risk, and these high-risk groups present an attractive focus for targeted control measures. To incorporate a risk structure for spillover in our model, we assumed that the high-risk group composed a fixed proportion (here *p*=0.10) of the population, and varied the rate ratio of zoonotic infection (*λ*_*H*_/*λ*_*L*_) between the high- and low-risk groups. Under no control, total incidence grows nonlinearly with increasing *R*, as in Fig. 2a, and also rises as the relative risk of spillover in the high-risk group increases (Fig. 6a). The latter effect is a simple reflection of increased total spillover in the population, as we treat *λ*_*L*_ as constant.

Considering different control strategies, we find that the broad hierarchy of strategies as described above is remarkably robust to heterogeneities in spillover risk, while the epidemiological parameters shape the potential benefit of targeted control. We first observe that untargeted control policies, such as general awareness campaigns aimed at reducing spillover in the whole population or human-to-human transmission, are unaffected by the defined risk groups (Fig. 6b & c). In contrast, a targeted strategy to reduce spillover, such as improved biosafety protocols among people who have high-risk contacts with animals, will have varying impact depending on the risk ratio (Fig. 6d), but as with universal spillover reduction (Fig. 6b), this impact does not exhibit any dependence on *R*. We also note that the overall incidence reduction appears lower for targeted control than for universal spillover control, but this is because this plot assumes equal reduction factors (*c* = 0.5) for all control measures; under this assumption, control measures are inevitably more impactful when applied to the whole population versus a high-risk group of just 10% of the population. Considering mixed strategies that combine targeted control of the high-risk spillover group with general control of human-to-human transmission (Fig. 6e-h), we see that the benefits of targeted control for total incidence reduction are greater for higher risk ratios, but this difference diminishes as *R* approaches 1 and human-to-human transmission dominates the epidemiology. As in Fig. 3b, the benefits of including human-to-human transmission controls depend on the delay before initiation. For longer delays the curves resemble the targeted spillover control in Fig. 6d for low values of *R*, since when *R* is low many transmission chains do not last long enough to be affected by reduced human-to-human transmission. In contrast, under joint programs with no delay (Fig. 6e), the benefits of control are seen immediately as *R* increases.

**Fig. 7:**
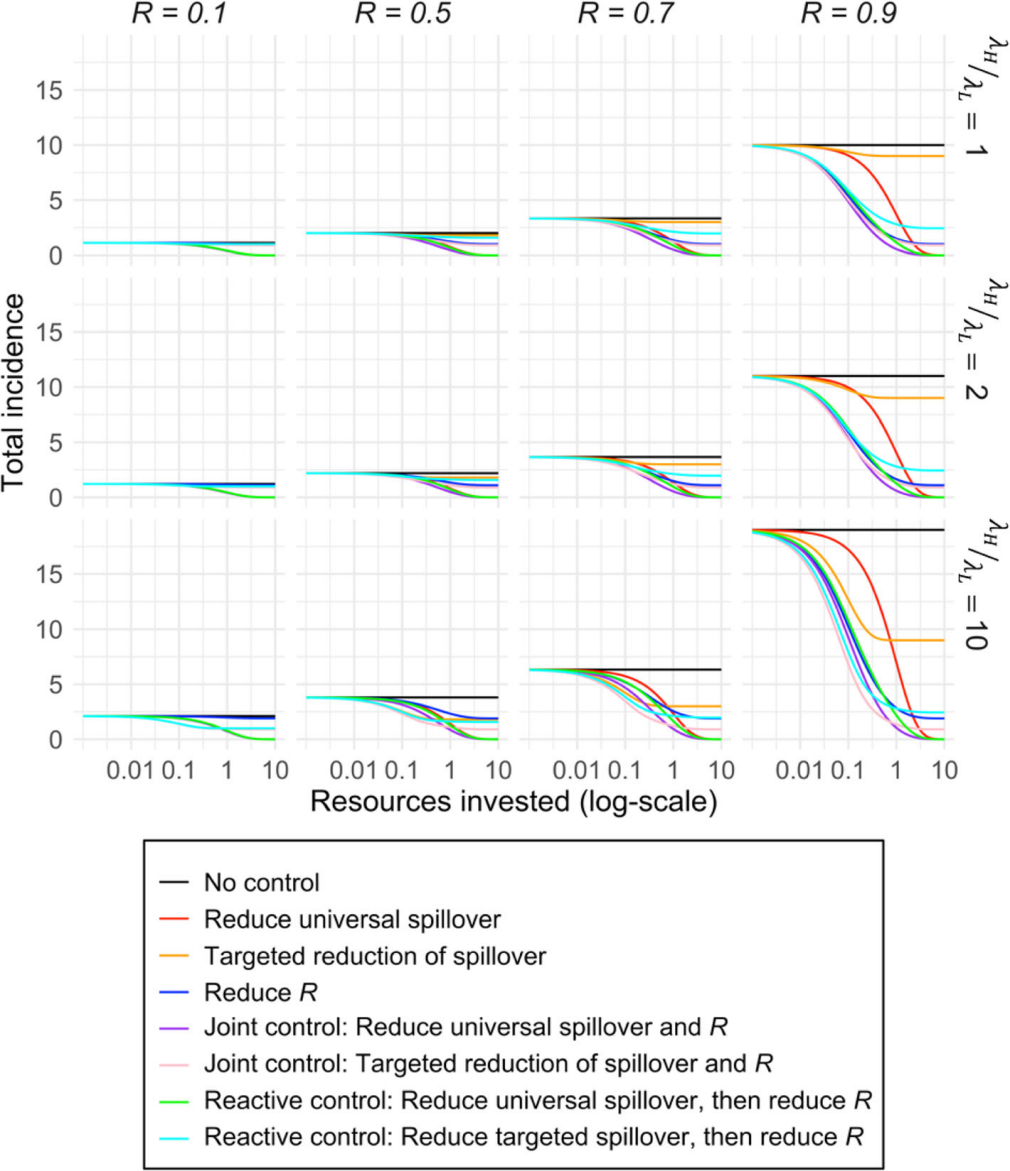
Impacts of different control measures with varying resource investment on the total incidence of a zoonotic infection with *R* (before control) between 0 and 1 with different ratios of high-to-low spillover rates. Columns (left to right) show increasing values of *R*. Rows (top to bottom) represent an increasing ratio of spillover rates in high-risk versus low-risk groups (*λ*_*H*_/*λ*_*L*_). The black lines indicate total incidence under no control. Colored lines represent the reduction in incidence for increasing resource investment. These scenarios were explored earlier in Fig. 4 but now include the added comparison of targeted versus universal spillover control for all strategies

Finally, we consider targeted control measures under different resource scenarios, to explore the possible benefits of efficiently reducing spillover in the high-risk group. Again, we see a tradeoff between strategies preferred at modest resource levels and those preferred when resources are not limiting (Fig. 7). Among strategies that only reduce spillover transmission (red and orange lines in Fig. 7), targeted control shows considerable benefits at low resource levels, particularly for high risk ratios and higher values of *R*. Yet targeted strategies are less effective at high resource levels, since they do not reduce spillover in the low-risk group, and hence can never reduce incidence to zero. A similar pattern is found for mixed strategies, where targeted joint or reactive approaches (i.e. high-risk spillover reduction followed by a switch to reducing human-to-human transmission once an outbreak is underway) are the most effective control policies at low resource levels, particularly when *R* > 0.5 and the risk ratio is high, but are incapable of reducing incidence to zero even at high levels of investment. In many other epidemiological settings, such as when *R* is 0.5 or lower and when risk ratios are near 1, any benefits to targeted control are imperceptible and tend to be outweighed by the disadvantages of allowing spillover to the low-risk group to continue unchecked.

## Discussion

Implementing efficient, cost-effective control measures is crucial for the control of emerging infectious disease, both to reduce the disease burden of human cases and to minimize the opportunities for pathogen adaptation, international spread, or other adverse events. However, for subcritical pathogens that exhibit low transmissibility among humans, it is not obvious whether control efforts should focus on reducing primary cases arising from spillover from external reservoirs or reducing secondary cases arising from human-to-human transmission. Using a simple mathematical model, we developed a theoretical framework to guide decisions about how to target resources under scenarios with different pathogen transmissibility and risk group structure. We focused on the relative impacts achievable in resource-constrained settings, as well as the maximum benefits that could be obtained when resource investment was high. Our work is framed in the context of zoonotic infections, where introductions arise via cross-species spillover from animal reservoirs, but our findings translate fully to other scenarios where outbreaks of subcritical pathogens are seeded by introductions from outside. This includes vaccine-preventable diseases such as measles in post-elimination settings where herd immunity has reduced *R* below 1, or pathogens such as Methicillin-resistant *Staphylococcus aureus* (MRSA) in hospitals that exhibit inefficient nosocomial transmission [39, 40, 69]. Geographic importation from endemic regions serves as ‘spillover’ for measles or COVID-19, introducing the pathogen into areas where it was previously eliminated or brought under control. Similarly, community introduction of MRSA into hospitals serves as the spillover mechanism prior to transmission within the hospital.

We found that the optimal focus of control measures for subcritical pathogens depends primarily on the human-to-human transmissibility of the pathogen, as summarized by the reproductive number *R* (Fig. 3). For pathogens with the lowest transmissibility among humans (*R* near zero; e.g. H7N9 avian influenza), measures to reduce zoonotic spillover are most effective. Thus for these zoonoses, strategies such as awareness campaigns to reduce contact with reservoir host animals or animals found dead, infection control in live animal markets, culling infected reservoir populations, and removing rodents from homes (Table 1) will be most effective in reducing human cases. For pathogens with greater transmissibility among humans (e.g. post-elimination measles, SARS-CoV-2, or Ebola), reducing human-to-human transmission becomes more effective. In such scenarios, preferred control methods will include providing personal protective equipment in high-risk settings such as hospitals, awareness campaigns to reduce unprotected contact with sick individuals, and strengthened surveillance for improved case tracking and faster case isolation. Of course, the strongest control strategies would act to reduce zoonotic spillover and human-to-human transmission at the same time, as with a protective vaccine. Where this option is not available (or is cost-prohibitive to deploy widely in advance of an outbreak), we found that a reactive strategy could achieve nearly the same effect without substantially greater investment. Such a strategy would have a baseline emphasis on reducing zoonotic spillover, but when a spillover or subsequent outbreak is discovered, the emphasis would shift locally to reducing human-to-human transmission. Strategies could include an awareness campaign that focuses on reducing interactions with known animal hosts, such as not touching dead animals in the forest, shifting to increased contact precautions and active surveillance to detect human cases quickly to reduce human-to-human transmission once an outbreak is detected [27].

Unsurprisingly, many existing control policies designed by public health professionals align broadly with the recommendations of our model. For pathogens with low *R* such as H7N9 avian influenza, our work advises an emphasis on preventing spillover. This is consistent with current public health control measures for H7N9 avian influenza, such as market disinfection or cessation of live poultry trade, which focus on reducing contact with birds and lowering risk of cross-species transmission [17–19]. In contrast, Lassa virus has a higher *R* value estimated near 0.7 and, thus, has a higher expected proportion of cases which arise from human-to-human transmission. Public health policy for Lassa fever has recently focused on preventing nosocomial transmission between humans [34–36]. In some settings, *R* changes through time due to shifts in population immunity or other factors, and priorities for disease control should change accordingly. For example, *R* for monkeypox has increased over the decades since the cessation of widespread smallpox vaccination around 1980 [28, 70], and the historic emphasis on spillover transmission should be re-examined in light of changing circumstances. Similarly, *R* for measles has risen as childhood vaccination rates have dropped [39, 71].

Public health systems frequently deal with resource constraints, in terms of finances and human or institutional capacity [72]. Exploring the effects of resource investment on the impacts of control on overall human incidence, our work illustrates potential trade-offs between higher cost effectiveness at low investment versus the ability to reduce incidence to zero at high investment (Fig. 4). At low investment levels, the best simple strategies are those which follow the priorities laid out above, i.e. focusing on spillover if *R* is low, or on human-to-human transmission if *R* is higher. However, as resource levels increase, the limitations of these simple priorities become clear, because strategies that omit the reduction of spillover cannot ever reduce human incidence to zero as they do not decrease the number of primary cases. Therefore, it is necessary to incorporate spillover reduction in any policy hoping to drive incidence to zero. When all types of interventions are assumed to be available with the same cost, then joint approaches that reduce both animal-to-human transmission and human-to-human transmission are most effective for a given resource level. When resources do not permit reduction of both transmission modes simultaneously, practitioners must decide which transmission mode is more important to control. Our analysis provides guidance as to which transmission method is best to control as a function of resource investment and *R*, accounting for possible differences in cost (Fig. 5). While further work is needed to characterize the cost-efficacy curves for control measures in particular systems, in order to implement this approach, this analysis provides a foundation for rational cost-benefit analysis to support disease control policy.

Zoonotic spillover risk is heterogeneous in the human population, since some groups have more frequent or riskier exposures to zoonotic reservoirs due to cultural or occupational factors [5]. Our analysis demonstrated that targeted spillover control in these high-risk groups offers the potential for markedly greater reductions in incidence, relative to control efforts spread across the entire population, when resources are limited. However, these targeted strategies are limited in impact as investment levels rise, since they don’t reduce the spillover rate in the low-risk group so they can never drive incidence to zero. If there is negligible risk in the low-risk group or if the low-risk group receives low-intensity control as a side benefit of the targeted control (for instance, via an awareness campaign focused on the high-risk group but available to all), then the targeted control strategy would remain the most efficient option. Ultimately, the desirability of targeting the high-risk spillover group depends on the epidemiological context (*R* value), resource level, and risk ratio between the risk groups.

It is important to recognize that there are often substantial challenges in identifying and quantifying risk factors for spillover, to support a rational decision about targeting. For zoonoses that spill over very infrequently and unpredictably, such as ebolaviruses, coronaviruses including SARS-CoV-2 and MERS, or some hantaviruses, the necessary data are hard to acquire and uncontrolled variation among spillover events can obscure patterns. In settings where the majority of individuals engage in potentially high-risk activities, yet spillover events are sporadic due to variation at other levels (e.g. in infection prevalence in the animal reservoir), it can be difficult to ascertain how the magnitude of risk is split across specific risk factors [2]. For example, in populations which nutritionally or economically rely on bushmeat, the majority of individuals can be exposed to multiple animal species through multiple modes of contact (e.g. hunting, food preparation, and cooking), which all present a potential risk of transmission [73]. The resulting difficulties in determining risk factors, and identifying distinct high-risk groups, can further complicate the implementation of targeted control measures.

Several caveats should be borne in mind in interpreting our analysis, which point to opportunities for further research. For the sake of clarity, our model is based on expected incidence levels under different scenarios, but stochastic variation can be large so individual outbreaks could differ substantially from our predictions. We also assumed stationarity (i.e. no changes in the model parameters through time), ignoring behavior change of affected populations or on-the-ground factors that can impede control measures [74]. We did not account for superspreading, or for variation in transmissibility across spatial or social contexts, both of which can have dramatic effects in the early phase of outbreaks [65, 75].

Our analysis incorporated heterogeneities in spillover risk but did not address different risk groups for on-going human-to-human transmission. Such a scenario could arise due to age-structured mixing or susceptibility, or from other risk factors such as occupational exposure in health-care settings, and could have important effects on outbreak dynamics [76, 77]. Factors giving rise to immune compromise, such as human immunodeficiency virus (HIV) infection, are another potential source of heterogeneity in both modes of transmission [78, 79]. The existence of distinct risk groups for human-to-human transmission would offer another important opportunity for targeted control measures and warrants further investigation. All of the above complexities could be addressed via more complex analytic methods, such as multitype branching processes, or via stochastic simulation analyses.

Our cost-benefit model is theoretical in nature and aims for simplicity rather than realism. We used a phenomenological model to represent diminishing returns on investment, but we do not properly account for complexities in scaling up control measures in space or time. For the reactive strategy, we assumed a clean and immediate switch between reducing spillover and reducing human-to-human transmission with no overlap and no lapse in control. In real-world settings, ramping up (or terminating) control measures is inevitably more complicated and a reactive strategy would likely entail delays and changing effectiveness through time. We also assumed there is no additional cost associated with switching strategies, but this is unlikely to be true due to the resources involved in initiating any program. While our theoretical analysis enabled a first exploration of the general cost-effectiveness of subcritical disease control, more intensive case studies are needed for specific pathogens.

## Conclusions

Subcritical zoonotic pathogens exist at the animal-human interface, and little policy guidance exists on the most effective ways to implement controls. In this study we present a framework to think systematically about controlling subcritical zoonoses, considering the relative importance of reducing animal-to-human spillover versus human-to-human transmission. Our work shows how the relative effectiveness of these strategies depends on epidemiological context and highlights a trade-off between cost-effectiveness at low resource levels and the potential to reduce incidence to zero as investment increases. Our findings illustrate core principles for evidence-based control of subcritical zoonoses and provide a foundation for integrative studies of particular systems to carry these ideas toward implementation.

## Supplementary information

**Additional file 1: Supplementary Section 1.** Derivation for the expected size of an outbreak under reactive control measures, for an implementation delay of k generations. **Supplementary Table 2.** A summary of equations and parameters for two group risk model.

## Data Availability

Not applicable.

## Abbreviations

CCHF: Crimean-Congo hemorrhagic fever
COVID-19: Coronavirus disease 2019
HIV: Human immunodeficiency virus
MERS: Middle East Respiratory Syndrome
MRSA: Methicillin-resistant *Staphylococcus aureus*
SARS-CoV: Severe acute respiratory syndrome coronavirus
SARS-CoV-2: Severe acute respiratory syndrome coronavirus 2
