# Supplementary material for: Controlling emerging zoonoses at the animal-human interface
Source: One Health Outlook. 2020 Sep 18;2:17. doi: 10.1186/s42522-020-00024-5 (PMC7550773; doi:10.1186/s42522-020-00024-5)
Supplement: Supplementary file 1 — Additional file 1: Supplementary Section 1. Derivation for the expected size of an outbreak under reactive control measures, for an implementation delay of k generations. Supplementary Table 2. A summary of equations and parameters for two group risk model. [file 42522_2020_24_MOESM1_ESM.docx]

**SUPPLEMENT**

**Derivation for the expected size of an outbreak under reactive control measures, for an implementation delay of *k* generations**

Under a reactive control strategy, human-to-human transmission is not controlled for *k* generations of transmission, representing delays in detecting that an outbreak is underway and ramping up implementation of new control measures. Prior to control, each infected case is expected to generate *R* cases in the next generation. Once controls on human-to-human transmission are in place, the expected number of cases drops from *R* to $c_{R}R$. Below, we have replaced $c_{R}$ with *c* for compactness in the derivation.

Let *k* be the number of transmission generations in which there is no control. The expected number of cases for the general case is derived by the sum of expected cases arising from uncontrolled transmission up to generation *k*, plus the sum of expected cases after generation *k* when control is in place, given by:

$$E\left( X \right)=\sum_{i=0}^{k} R^{i}+\sum_{i=1}^{\infty} R^{k}{(cR)}^{i}$$

$$=\sum_{i=0}^{k} R^{i}+\frac{1}{c^{k}}\sum_{i=1}^{\infty} c^{k}R^{k}{(cR)}^{i}$$

$$=\sum_{i=0}^{k} R^{i}+\frac{1}{c^{k}}\left[ \sum_{i=1}^{\infty} \left( cR \right)^{k+i}+\sum_{i=0}^{k} \left( cR \right)^{i} \right]-\frac{1}{c^{k}}\left[ \sum_{i=0}^{k} \left( cR \right)^{i} \right]$$

$$=\sum_{i=0}^{k} R^{i}+\frac{1}{c^{k}}\left[ \sum_{i=0}^{\infty} \left( cR \right)^{i} \right]-\left[ \sum_{i=0}^{k} \frac{1}{c^{k}}\left( cR \right)^{i} \right]$$

$$=\frac{1}{c^{k}}\left[ \frac{1}{1-cR} \right]+\sum_{i=0}^{k} \left[ R^{i}-\frac{1}{c^{k}}\left( cR \right)^{i} \right]$$

$$=\frac{1}{c^{k}}\left[ \frac{1}{1-cR} \right]+\sum_{i=0}^{k} \left[ R^{i}\left( 1-\frac{c^{i}}{c^{k}} \right) \right]$$

Table S1: **A summary of equations and parameters for two group risk model.**

| **Control measure** | **Equation** |
| --- | --- |
| No control | $I_{c}=\frac{p\lambda_{H} +(1-p)\lambda_{L}}{1-R}$ |
| Reduction of general spillover | $I_{c}=\frac{c_{\lambda}\left( p\lambda_{H} +(1-p)\lambda_{L} \right)}{1-c_{R}R}$ |
| Reduction of high-risk spillover | $I_{c}=\frac{c_{H}p\lambda_{H} +(1-p)\lambda_{L}}{1-R}$ |
| Reduction of *R* | $I_{c}=\frac{p\lambda_{H} +(1-p)\lambda_{L}}{1-c_{R}R}$ |
| Reduction of universal spillover and *R* | $I_{c}=\frac{c_{\lambda}\left( p\lambda_{H} +(1-p)\lambda_{L} \right)}{1-c_{R}R}$ |
| Reduction of high-risk spillover and *R* | $I_{c}=\frac{c_{H}p\lambda_{H}+\left( 1-p \right)\lambda_{L}}{1-c_{R}R}$ |
| Reduction of general spillover, then reduction of *R* | $I_{c}=c_{\lambda}\left( p\lambda_{H}+\left( 1-p \right)\lambda_{L} \right)\left( \frac{1}{1-c_{R}R}+\left( 1-c_{R} \right)\sum_{i=1}^{k} R^{i} \right)$ |
| Reduction of high-risk spillover, then reduction of *R* | $I_{c}=\left( c_{H}p\lambda_{H}+\left( 1-p \right)\lambda_{L} \right)\left( \frac{1}{1-c_{R}R}+\left( 1-c_{R} \right)\sum_{i=1}^{k} R^{i} \right)$ |
| Reduction factor of general spillover | $c_{\lambda}=e^{-\alpha r}$ |
| Reduction factor of *R* | $c_{R}=e^{-r}$ |
| Reduction factor of high-risk spillover | $c_{H}=e^{-\alpha r/p}$ |
